# Predictors and surgical outcome of hemorrhagic metastatic brain malignancies

**DOI:** 10.1007/s11060-024-04714-2

**Published:** 2024-05-27

**Authors:** Laurèl Rauschenbach, Pia Kolbe, Adrian Engel, Yahya Ahmadipour, Marvin Darkwah Oppong, Alejandro N. Santos, Sied Kebir, Celia Dobersalske, Björn Scheffler, Cornelius Deuschl, Philipp Dammann, Karsten H. Wrede, Ulrich Sure, Ramazan Jabbarli

**Affiliations:** 1 Department of Neurosurgery and Spine Surgery, University Hospital Essen, Hufelandstrasse 55, 45147 Essen, Germany; 2 DKFZ Division of Translational Neurooncology at the West German Cancer Center (WTZ), University Hospital Essen, Essen, Germany; 3 German Cancer Consortium (DKTK), partner site Essen/Düsseldorf, University of Duisburg-Essen, and German Cancer Research Center (DKFZ), Heidelberg, Germany; 4 Center for Translational Neuroscience and Behavioral Science (C-TNBS), University of Duisburg-Essen, Essen, Germany; 5 Division of Clinical Neurooncology, Department of Neurology, University Hospital Essen, Essen, Germany; 6 Institute for Diagnostic and Interventional Radiology, University Hospital Essen, Essen, Germany

**Keywords:** Brain metastasis, Intracerebral hemorrhage, Hemorrhagic brain tumor, Outcome

## Abstract

**Purpose:**

Intracerebral metastases present a substantial risk of tumor-associated intracerebral hemorrhage (ICH). This study aimed to investigate the risk of hemorrhagic events in brain metastases (BM) from various primary tumor sites and evaluate the safety and outcomes of surgical tumor removal.

**Methods:**

A retrospective, single-center review of medical records was conducted for patients who underwent BM removal between January 2016 and December 2017. Patients with hemorrhagic BM were compared to those with non-hemorrhagic BM. Data on preoperative predictors, perioperative management, and postoperative outcomes were collected and analyzed.

**Results:**

A total of 229 patients met the inclusion criteria. Melanoma metastases were significantly associated with preoperative hemorrhage, even after adjusting for confounding factors (*p* = 0.001). Poor clinical status (*p* = 0.001), larger tumor volume (*p* = 0.020), and unfavorable prognosis (*p* = 0.001) independently predicted spontaneous hemorrhage. Importantly, preoperative use of anticoagulant medications was not linked to increased hemorrhagic risk (*p* = 0.592). Surgical removal of hemorrhagic BM, following cessation of blood-thinning medication, did not significantly affect intraoperative blood loss, surgical duration, or postoperative rebleeding risk (*p* > 0.096). However, intra-tumoral hemorrhage was associated with reduced overall survival (*p* = 0.001).

**Conclusion:**

This study emphasizes the safety of anticoagulation in patients with BM and highlights the safety of neurosurgical treatment in patients with hemorrhagic BM when blood-thinning medication is temporarily paused. The presence of intra-tumoral hemorrhage negatively impacts survival, highlighting its prognostic significance in BM patients. Further research with larger cohorts is warranted to validate these findings and elucidate underlying mechanisms.

**Supplementary Information:**

The online version contains supplementary material available at 10.1007/s11060-024-04714-2.

## Introduction

Intracerebral metastases represent the predominant form of brain malignancy in adults, with lung cancer, breast cancer, colorectal cancer, melanoma, and renal cell carcinoma posing the highest risk of metastasizing to the brain [1]. With advancements in high-resolution imaging and improved management of extracranial tumor diseases, there has been a notable rise in the incidence of brain metastases (BM) [2].

Historically, BM have been associated with an elevated risk of tumor-associated intracerebral hemorrhage (ICH), which can have devastating consequences [3]. Among those, BM originating from melanoma, kidney carcinoma, and hepatocellular carcinoma [4–8] are believed to carry an increased risk of ICH [9–13]. Despite numerous studies addressing the safety of anticoagulation in these patients, many exclude those who have undergone neurosurgical tumor resection.

Presently, the surgical removal of BM is both feasible and safe, but there remains a lack of research focusing on the surgical treatment of hemorrhagic BM or those with a higher disposition to hemorrhage due to their primary origin [14]. Consequently, it remains unclear whether patients with BM from different tumor entities are more susceptible to perioperative hemorrhagic complications than others.

This study aims to investigate the risk of hemorrhagic events in BM originating from distinct tumor entities and to assess the safety of surgical tumor removal in these cases.

## Methods

### Study design

The medical records of consecutive patients who underwent intracerebral tumor removal at our tertiary department between January 01, 2016, and December 31, 2017, were retrospectively reviewed and individuals with histologically confirmed BM were identified. This study did not require individual patient consent since data collection was performed retrospectively. All patient identifiers had been removed from the data set and no personal information on any subject or medical care provider could be obtained. The study was conducted in accordance to the principles expressed in the Declaration of Helsinki and the guidelines of an approving local research ethics committee (approval number: 18-8475-BO) and was registered in a national database of clinical studies (registration number: DRKS-00019808).

### Treatment work-flow

Indication for neurosurgical intervention was made individually for each patient according to an interdisciplinary local tumor board or after interdisciplinary discussion between neurosurgical and oncology physicians on duty in case of emergency. Postoperative care of all patients was provided in a neuro-intensive care unit. Patients underwent cranial, thoracic, and abdominal CT imaging and brain MRI preoperatively, and cranial CT imaging postoperatively. Laboratory chemistry was performed immediately before surgery and after surgery to rule out pre- or postoperative coagulation disorders. The values of activated partial thromboplastin time (aPTT), prothrombin time (PT), international normalized ratio (INR), platelet count, hemoglobin (Hb), and hematocrit (Hct) were determined. If any abnormalities were found, supplements were administered before or after surgery to achieve normal conditions. For patients receiving blood-thinning medications, the medication was paused before the surgery according to the following procedure: The use of platelet aggregation inhibitors such as aspirin and clopidogrel was paused seven days before surgery, the application of unfractionated heparin or low-molecular-weight heparin one day before surgery, the administration of direct oral anticoagulants was paused two to three days before the operation, and warfarin was replaced by unfractionated heparin or low-molecular-weight heparin with bridging. Thromboprophylaxis was given to all patients, usually with low-molecular-weight heparins, unless renal insufficiency was present, in which case unfractionated heparin was used. Prophylaxis was paused on the day before surgery and the day of surgery. All intraoperatively resected tissues underwent neuropathologic diagnosis, including immunohistochemistry, to determine the primary cancer site.

### Inclusion and exclusion criteria

Participation in the study necessitated the availability of the majority of clinical baseline data, as well as all neuropathological and neuroradiological data. Follow-up data were collected for cases where available. Only patients with sufficient coagulation (cessation of the medication according to the above-mentioned work-flow and normalized parameters in the blood test immediately before surgery) were included. Lymphoma disease was excluded from this study. Only patients who underwent complete tumor resection according to the surgical report were included, while patients with incompletely resected metastases were excluded. Patients with BM and ICH attributable to trauma or cerebrovascular diseases like aneurysms or arteriovenous malformations were excluded.

### Clinical data

Baseline clinical characteristics were identified using patient charts, encompassing admission records, anesthesia records, surgical notes, neuropathology reports, intensive or intermediate care unit notes, discharge letters, and follow-up examination reports. These characteristics included age at primary tumor diagnosis and BM diagnosis, gender, tumor diagnosis, comorbidities, prior use of anticoagulation and/or steroids, previous treatment with irradiation and/or chemotherapy, laboratory findings, length of surgery for BM removal, intraoperative blood loss, incidence and severity of postoperative bleeding associated with the resection cavity, and overall survival.

Clinical performance was evaluated based on the Karnofsky Performance Status (KPS) scale. A KPS score of 70 or higher indicated favorable performance, while a score below 70 indicated unfavorable performance. Prognosis was determined using the Recursive Partitioning Analysis (RPA) and the disease-specific Graded Prognostic Assessment (dsGPA) classification. For both classification tools, a score of 2 or lower was classified as favorable and a score greater than 2 was classified as unfavorable. Comorbidity assessment was conducted using the Charlson Comorbidity Index (CCI), with a score of 10 or lower considered favorable and a score higher than 10 considered unfavorable.

### Imaging data

Preoperative CT scans of the thorax and abdomen were employed to evaluate the presence and quantity of extracranial metastases, as well as to assess the extent of local control of the primary tumor. Preoperative CT and MRI scans of the head were meticulously scrutinized to determine the number and precise locations of BM, the depth of BM within the brain, and to identify any tumor-associated ICH. Quantification of tumor volume was conducted utilizing iPlan Net software by BrainLab AG (Germany) and 3D Slicer software by The Brigham and Women’s Hospital, Inc. (USA). In the case of intra- and extra-tumoral bleeding, the hemorrhage and tumor were considered together as one lesion. The final volume measurements (in cm^3^) were derived from calculated mean values obtained through both segmentation tools. The depth of metastases was measured (in mm) by determining the shortest distance between the cortex and the surface of the metastasis. Both smaller hemorrhages within the tumors and without contact to the surrounding brain parenchyma as well as larger hemorrhages with contact to the brain parenchyma were detected. The diagnosis of hemorrhage was established based on the presence of lesion-associated blood fluid levels observed on MRI and/or CT scans, along with susceptibility artifacts noted on iron-sensitive MRI sequences, such as susceptibility-weighted imaging or T2*-weighted gradient-echo imaging. Corresponding hyperdensity on non-enhanced CT ≥ 85 Hounsfield Units was interpreted as calcification and absence of hemorrhage. Based on this algorithm, acute, subacute, and chronic bleeding events were recorded.

### Statistical analysis

Data analysis was conducted using SPSS-22, with visualization facilitated by PRISM-9. Univariate analyses were undertaken to identify predictors of outcome. For dichotomized variables, the Chi-Square test (for sample sizes greater than 5) or the Fisher exact test (for sample sizes equal to or less than 5) was applied. Odds ratios (OR) and 95% confidence intervals (95%CI) were computed to evaluate the degree of association between specific factors and the occurrence of hemorrhage associated with brain metastases. Continuous variables were assessed using either the Student’s t-Test (for normally distributed data) or the Mann-Whitney-U test (for non-normally distributed data), with normal distribution tested via the Shapiro-Wilk test. Multivariate analyses were executed utilizing a binary regression model, incorporating associations identified in the univariate analyses. To evaluate the impact of hemorrhage associated with BM on patients’ overall survival, univariate and multivariate Cox regression analyses were conducted to determine the hazard ratio (HR). Survival data were depicted using a Kaplan-Meier curve, with survival disparities among patient groups assessed through the log-rank test. To investigate the correlation between survival and the dsGPA score, a Pearson correlation analysis was performed, with “r” describing the Pearson’s correlation coefficient. Statistical significance for all analyses was established at *p* < 0.05, with hypothesis testing conducted on a two-sided basis.

## Results

### Study cohort

A total of 252 patients were screened for eligibility and 23 patients were subsequently removed from the study. Finally, 229 patients met all inclusion criteria and were referred to further analyses. At the time of diagnosis of the underlying tumor disease, the patients were 58 (± 12) years old. Half of the patients (*N* = 115; 50.2%) were female. Almost all patients suffered from other diseases beyond their tumor diagnosis and the median comorbidity index was 8 (IQR = 5–10). At an average age of 62 (± 11) years, the patients underwent brain surgery for metastatic tumor removal. The interval between the initial diagnosis of the primary disease and brain tumor surgery averaged 38 (± 67) months. Almost half of the patients (*N* = 113, 49.3%) suffered from lung cancer, predominantly non-small cell lung cancer (*N* = 99, 43.2%). Less frequently but still often, patients suffered from breast cancer (*N* = 24, 10.5%) or melanoma (*N* = 24, 10.5%). At the time of brain tumor surgery, patients revealed a median KPS score of 90 (IQR = 80–90). In the majority of cases (*N* = 140, 61.1%) subjects showed extracranial metastases. Accordingly, the patients revealed a median RPA score of 2 (IQR = 2–2). Most patients revealed singular BM, but a relevant subset of patients had multiple BM (*N* = 96, 41.9%). In these cases, most patients (*N* = 76, 82.3%) suffered from two lesions, and a small proportion (*N* = 20, 20.8%) from three lesions. The operated tumors showed an average tumor volume of 17 cm^3^ (± 19 cm^3^) and a distance to the cerebral cortex of 19 mm (±10 mm). Most lesions were located in the frontal lobe (*N* = 97, 42.4%), followed by metastases to the cerebellum (*N* = 60, 26.2%). Prior to BM removal, most patients (*N* = 144, 62.9%) received systemic therapy, and a small proportion of tumors underwent prior radiation (*N* = 47, 20.5%). At the time of brain tumor surgery, the majority of patients (*N* = 187, 81.7%) were receiving steroid treatment for brain edema. Survival data were available for a large proportion of the cohort. Detailed data is presented in Table 1.

**Table 1:** Demographic, anatomic and clinical characteristics

Characteristic	Frequency
Total number of patients, N (%)	229 (100)
Age:	
• Diagnosis primary tumor, years, mean ± SD	58.0 ± 11.6
• Diagnosis secondary tumor, years, mean ± SD	61.5 ± 11.1
Female sex, N (%)	115 (50.2)
Tumor diagnosis:	
• SCLC/NSCLC, N (%)	113 (49.3)
• Melanoma, N (%)	24 (10.5)
• Breast cancer, N (%)	24 (10.5)
• GIT cancer, N (%)	17 (7.4)
• Urogenital cancer, N (%)	17 (7.4)
• CUP syndrome, N (%)	15 (6.6)
• Miscellaneous^#^, N (%)	8 (3.5)
• Renal cancer, N (%)	7 (3.1)
• Head/Neck cancer, N (%)	4 (1.7)
Clinical performance:	
• KPS, median (IQR)	90 (80– 90)
• RPA score, median (IQR)	2 (2– 2)
• dsGPA score, median (IQR)	2 (1.75– 3.00)
• CCI, median (IQR)	8 (5– 10)
BM multiplicity:	
• 1 BM, N (%)	133 (58.1)
• 2 BM, N (%)	76 (33.2)
• 3 BM, N (%)	20 (8.7)
BM features:	
• Volume, cm^3^, mean ± SD	17.3 ± 19.0
• Depth, mm, mean ± SD	18.6 ± 9.9
• Localization:	
- Frontal lobe, N (%)	97 (42.4)
- Parietal lobe, N (%)	24 (10.5)
- Temporal lobe, N (%)	27 (11.8)
- Occipital lobe, N (%)	21 (9.2)
- Cerebellum, N (%)	60 (26.2)
Prior treatment:	
• Systemic treatment, N (%)	144 (62.9)
• BM irradiation, N (%)	47 (20.5)
• Steroid treatment, N (%)	187 (81.7)
• Anticoagulation, N (%)	54 (23.6)

The collected parameters of the entire cohort are presented. *Abbreviations* BM, brain metastasis; CCI, Charlson Comorbidity Index; CUP, cancer of unknown primary; dsGPA, diagnosis-specific Graded Prognostic Assessment; GIT, gastrointestinal tract; IQR, interquartile range; KPS, Karnofsky Performance Scale; N, number of patients; NSCLC, non-small-cell lung cancer; RPA, Recursive Partitioning Analysis; SCLC, small-cell lung cancer; SD, standard deviation; #, sarcoma (*N* = 5), thyroid carcinoma (*N* = 1), hepatocellular carcinoma (*N* = 1), basal cell carcinoma (*N* = 1)

### Predictors of preoperative BM-associated hemorrhage

**Fig. 1:**
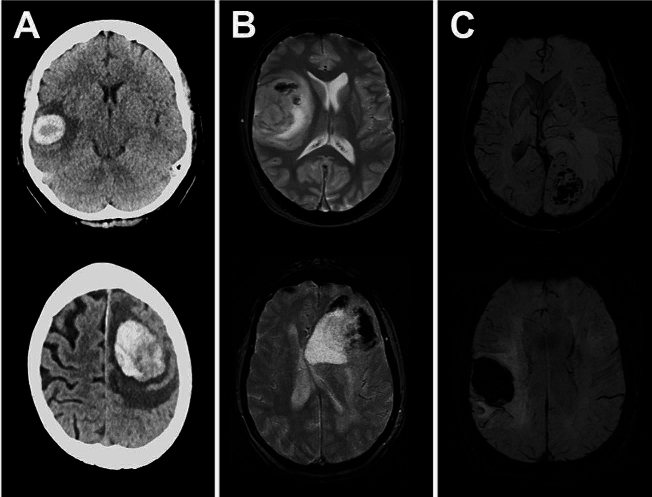
Exemplary cases of patients with hemorrhagic BM. Shown are 6 different patients with hemorrhagic brain metastases, using (**A**) CT imaging, (**B**) MRI / GRE-T2* imaging, and (**C**) MRI / SWI imaging. The axial slices are shown

In a large subgroup of patients (*N* = 54, 23.6%), metastasis-associated hemorrhage was evident on preoperative imaging. Exemplary cases are illustrated in Fig. 1. In these 54 cases, most patients suffered from metastases of lung carcinoma (*N* = 23, 42.6%) or melanoma (*N* = 14, 25.9%), while the incidence of breast cancer metastasis (*N* = 1, 1.9%) was very low. For all entities, melanoma (*N* = 14, 58.3%) showed the highest incidence of BM-associated hemorrhage, and diagnosis of melanoma metastasis was associated with tumor-related bleeding in univariate analysis (OR = 5.78, 95%CI = 2.39–13.95, *p* = 0.001). On the other hand, the overall incidence of hemorrhage for breast cancer patients was low (*N* = 1, 4.35%) and diagnosis of breast cancer was associated with the lack of bleeding in univariate analysis (OR = 0.13, 95%CI = 0.02–1.00, *p* = 0.034). Moreover, poor clinical status, as measured by a KPS score of < 70 (OR = 30.26, 95%CI = 3.69–248.13, *p* = 0.001), and impaired prognosis, as measured by an RPA score of > 2 (OR = 30.26, 95%CI = 3.69–248.13, *p* = 0.001), were associated with a higher risk of bleeding events in the univariate analysis. Furthermore, larger metastases revealed a higher risk for tumor-associated hemorrhage (*p* = 0.044). One-fourth of all patients (*N* = 57, 24.9%) were treated preoperatively with blood-thinning medications, but there was no association between preoperative use of these drugs and the occurrence of tumor-associated bleeding (OR = 0.90; 95%CI = 0.518–1.563; *p* = 0.426). Table 2 illustrates the results of univariate analyses and highlights the predictors for BM-related hemorrhage.

**Table 2:** Univariate analysis of predictors for BM-related hemorrhage

Parameter	BM with hemorrhage (*N* = 54)	BM w/o hemorrhage (*N* = 175)	*p*-value	OR	95%CI
Age in years, mean ± SD	61.5 ± 13.6	61.5 ± 10.3	0.984 ^a^	N/A	N/A
Female sex, N (%)	27 (50.0)	88 (50.3)	0.999 ^b^	0.99	0.54– 1.82
**Tumor diagnosis**:					
• SCLC/NSCLC, N (%)	23 (42.6)	90 (51.4)	0.279 ^b^	0.7	0.38– 1.30
• Melanoma, N (%)	14 (25.9)	10 (5.71)	**0.001** ^b^	5.78	2.39– 13.95
• Breast cancer, N (%)	1 (1.9)	22 (12.6)	**0.034** ^b^	0.13	0.02– 1.00
• GIT cancer, N (%)	5 (9.3)	12 (6.9)	0.768 ^b^	1.39	0.47– 4.13
**Clinical performance**:					
• KPS < 70, N (%)	8 (14.8)	1 (0.6)	**0.001** ^b^	30.26	3.69– 248.13
• RPA > 2, N (%)	8 (14.8)	1 (0.6)	**0.001** ^b^	30.26	3.69– 248.13
• dsGPA > 2, N (%)	19 (35.2)	59 (33.7)	0.870 ^b^	1.07	0.56– 2.03
Comorbidity:					
• CCI > 10, N (%)	11 (20.4)	33 (37.7)	0.844 ^b^	1.1	0.51– 2.36
BM multiplicity, N (%)	23 (42.6)	73 (41.7)	0.999 ^b^	1.04	0.56– 1.92
**BM features**:					
• Volume, cm^3^, mean ± SD	21.7 ± 26.2	15.7 ± 15.9	**0.044** ^a^	N/A	N/A
• Depth, mm, mean ± SD	19.7 ± 8.5	18.3 ± 10.3	0.349 ^a^	N/A	N/A
• Localization:					
- Frontal lobe, N (%)	26 (48.1)	71 (40.6)	0.347 ^b^	1.36	0.74– 2.51
- Parietal lobe, N (%)	6 (14.8)	18 (10.3)	0.999 ^b^	1.01	0.41– 2.90
- Temporal lobe, N (%)	7 (13.0)	21 (12.0)	0.999 ^b^	1.09	0.44– 2.73
- Occipital lobe, N (%)	3 (5.6)	17 (9.7)	0.421 ^b^	1.75	0.53– 5.74
- Cerebellum, N (%)	12 (22.2)	48 (27.4)	0.485 ^b^	1.23	0.71– 2.15
**Prior treatment**:					
• Systemic treatment, N (%)	29 (53.7)	115 (65.7)	0.147 ^b^	0.61	0.33– 1.12
• BM irradiation, N (%)	8 (14.8)	39 (22.3)	0.256 ^b^	0.61	0.26– 1.39
• Steroid treatment, N (%)	44 (81.4)	143 (81.7)	0.999 ^b^	0.99	0.45– 2.16
• Anticoagulation, N (%)	15 (27.8)	42 (24.0)	0.592 ^b^	1.22	0.61– 2.43

Univariate analysis of demographic, anatomic, and clinical factors for association with BM related hemorrhage. Note All parameters refer to the examinations immediately before surgery. All significant factors are highlighted in bold style. *Annotations*^a^ Student’s t-test or Mann-Whitney-U test; ^b^ Chi-Square test or Fisher exact test. *Abbreviations* BM, brain metastasis; CCI, Charlson Comorbidity Index; dsGPA, diagnosis-specific Graded Prognostic Assessment; GIT, gastrointestinal tract; KPS, Karnofsky Performance Scale; N, number of patients; NSCLC, non-small-cell lung cancer; OR, odds ratio; RPA, Recursive Partitioning Analysis; SCLC, small-cell lung cancer; SD, standard deviation; w/o, without

Predictors of tumor-associated bleeding identified in univariate analyses were submitted to multivariate analysis. Since the KPS score and the RPA score classified the patient population identically, only the KPS score was included in the analysis due to redundancies. At this point, we have opted for the KPS over the RPA score due to its widespread acceptance and utilization in recording the overall patient status. The analysis confirmed that melanoma metastasis (aOR = 5.52, 95%CI = 2.19–13.95, *p* = 0.001), poor clinical status (aOR = 60.07, 95% CI = 5.41–666.62, *p* = 0.001), and large tumor volume (aOR = 3.17, 95%CI = 1.20–8.41, *p* = 0.020) were independent predictors of bleeding. The results of the multivariate analysis can be seen in Table 3.

**Table 3:** Multivariate analysis of predictors for BM-related hemorrhage

Parameter	*p*-value	aOR	95%CI
Tumor diagnosis: melanoma	0.001	5.52	2.19–13.95
Clinical performance: KPS score < 70	0.001	60.07	5.41–666.62
Tumor feature: volume > 5cm^3^	0.020	3.17	1.20–8.41

Multivariate analysis of selected variables for independent association with BM-related hemorrhage. Note All variables that reached an alpha level of < 0.05 in the univariate analysis were included in this multivariate analysis. Due to redundancies, only the KPS and not the RPA score was included. All significant factors are highlighted in bold style. *Abbreviations* aOR, adjusted odds ratio; BM, brain metastasis; KPS, Karnofsky Performance Scale

### Postoperative hemorrhage events

Preoperatively, normotensive coagulation was achieved in all patients by pausing blood-thinning drugs and/or substituting procoagulant medications. The patients consistently revealed normal coagulation parameters by laboratory chemistry and the mean values for the activated partial thromboplastin time (aPTT) were 24.59 ± 2.51 s, for the prothrombin time (PT) 101.40 ± 13.50%, for the international normalized ratio (INR) 1.42 ± 6.37, for the platelet counts 280.75 ± 108.7 / nL, for the amount of hemoglobin (Hb) 13.0 ± 2.08 g / dL, and for the hematocrit (Hct) 0.39 ± 0.10%. Univariate analysis of the respective parameters showed the same laboratory constellation for patients with or without hemorrhagic BM. Data is illustrated in the Supplementary Table 1.

**Fig. 2:**
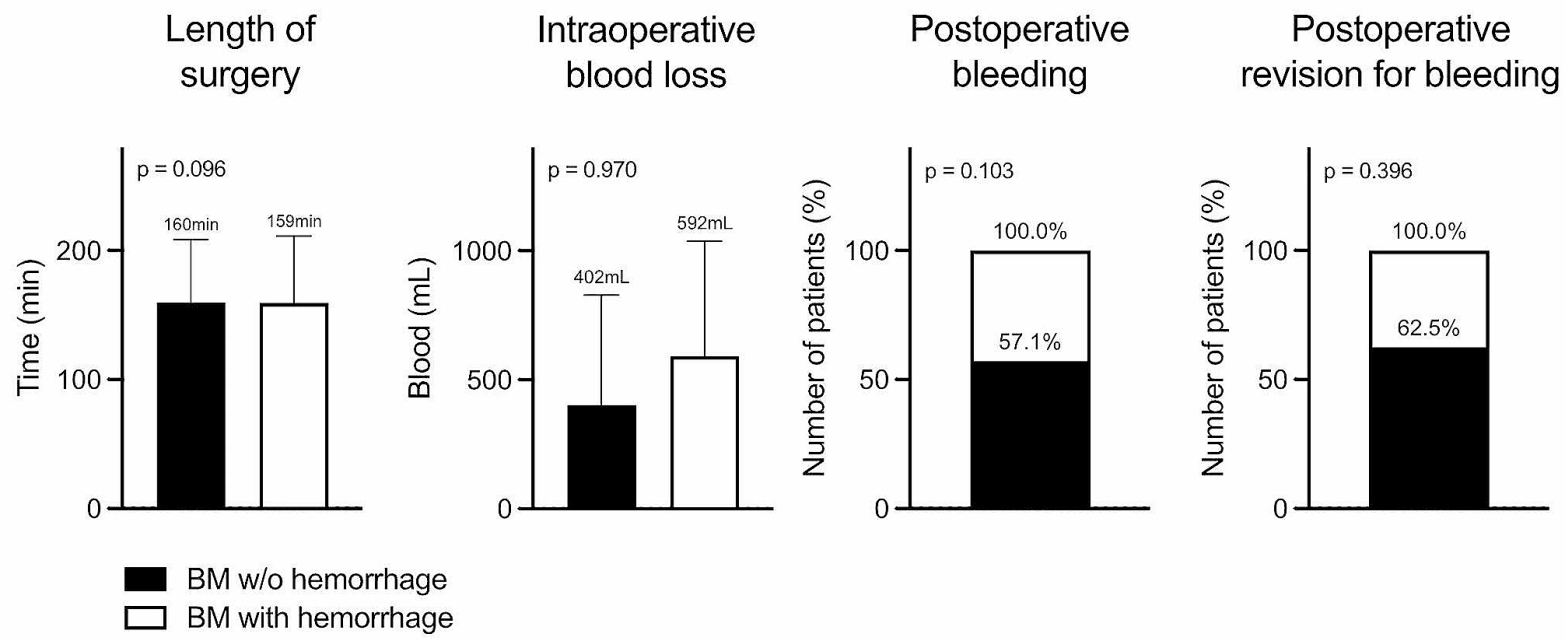
Intra- and postoperative complications. The study compares patients with and without hemorrhagic BM across several parameters. These include the duration of the operation (in minutes), intraoperative blood loss (in milliliters), the incidence of postoperative hemorrhages (as a percentage), and the frequency of revisions necessitated by postoperative hemorrhages (as a percentage). Each parameter was analyzed using a Student’s t-test, and the corresponding p-value is displayed in the respective diagram. *Abbreviations* BM, brain metastasis; w/o, without

Data on intraoperative blood loss were available for 82 patients. Comparative analysis showed that hemorrhagic brain metastases did not cause increased blood loss during surgery (*p* = 0.970). This was also applied for the length of surgery, with hemorrhagic brain metastases not taking a longer time for removal (*p* = 0.096). Postoperatively, 14 patients experienced rebleeding, but hemorrhagic brain metastases were not more likely to result in rebleeding (*p* = 0.103) or even rebleeding that required revision (*p* = 0.396). Data is illustrated in Fig. 2.

### Postoperative outcome

**Fig. 3:**
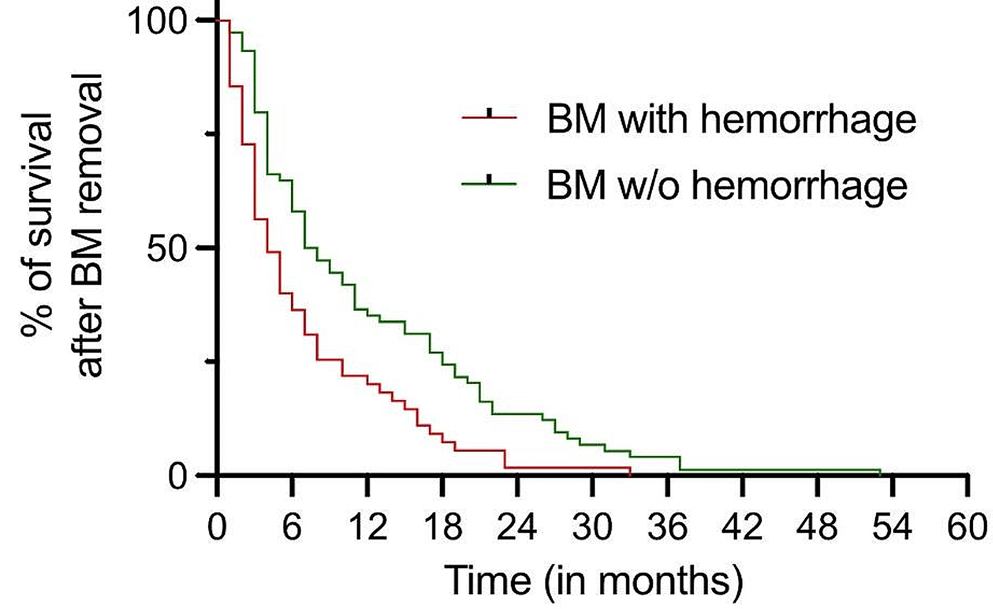
Overall survival following metastasectomy in patients with and without hemorrhagic BM. Patients without hemorrhagic BM are represented by the green line, while patients with hemorrhagic BM are depicted by the red line. *Abbreviations* BM, brain metastasis; w/o, without

As part of our internal quality control procedures, we examined the correlation between the dsGPA scores and survival outcomes. Utilizing Pearson’s correlation analysis, we identified a robust correlation between the dsGPA score and survival (*r* = 0.359; *p* < 0.001). Notably, patients with higher dsGPA scores exhibited significantly longer survival periods compared to those with lower scores. To evaluate the extent to which a hemorrhage induced by BM influences the overall survival of patients, a univariate Cox regression analysis was conducted. Survival time data were available for a total of 139 out of 229 patients included in the study. The results indicate that the presence of a hemorrhage is significantly associated with patient survival (HR = 1.47; 95% CI = 1.03–2.09; *p* = 0.03). Multivariate Cox regression analysis investigated the following items: patient age at the time of surgery, number of BM, diagnosis of primary disease (SCLC/NSCLC, melanoma, breast cancer, GIT cancer), preoperative KPS score, and presence of hemorrhage. Analysis revealed BM multiplicity (aHR = 1.60; 95% CI = 1.10–2.20; *p* = 0.01) and BM-associated hemorrhage (aHR = 1.53; 95% CI = 1.04–2.24; *p* = 0.03) as independent and significant predictors for dismal survival. The collected data were visualized using a Kaplan-Meier curve, and the median survival was subsequently determined. Patients exhibiting signs of hemorrhage had a significantly shorter median survival of 4 months post-metastasectomy compared to patients without signs of hemorrhage, who revealed a median survival of 7.5 months. Additionally, the log-rank test conducted shows a statistically significant difference between the two patient groups (*p* = 0.001). The results are depicted in Fig. 3.

## Discussion

Cerebral metastasizing represents a significant clinical challenge in oncology, with increasing incidence of BM due to improved diagnostic techniques and prolonged survival of patients with systemic cancer [2]. Intratumoral hemorrhage is a commonly observed phenomenon in BM; however, there is a notable lack of literature elucidating the predictors and consequences of hemorrhagic events, especially concerning their management through surgical intervention. Thus, uncertainty does exist regarding the surgical removal of hemorrhagic metastases or lesions prone to hemorrhage, like melanoma, kidney carcinoma, or hepatocellular carcinoma. This study aimed to address this gap by investigating the incidence of hemorrhagic BM from distinct primary tumor sites, the risk factors for preoperative hemorrhage, and the impact of surgical treatment on postoperative outcomes.

The findings of this study corroborate existing literature suggesting that certain primary tumors, notably melanoma, are associated with a higher risk of hemorrhagic BM [11, 15, 16]. Patients with melanoma metastases were significantly more likely to present with preoperative hemorrhage compared to other primary tumor types and the association remained significant even after controlling for confounding factors in multivariate analysis, suggesting an independent relationship between melanoma origin and hemorrhagic events. Furthermore, in line with prior research findings, this study identified poor clinical status, as measured by the KPS score, as an independent predictor of spontaneous hemorrhage in patients with BM [17]. Additionally, unfavorable clinical prognosis, as assessed by the RPA score, along with larger tumor volume, were found to be independent predictors of spontaneous hemorrhage in patients with BM. Taken together all factors likely reflect advanced disease burden and aggressive tumor biology, predisposing to vascular instability and hemorrhagic complications [18].

Importantly, the use of anticoagulant medications was not associated with an increased incidence of hemorrhagic BM, indicating that these medications can be safely administered to patients with metastasized cancer disease. This finding is consistent with previous large-scale studies on the safety of anticoagulation in BM patients and underscores the importance of effectively managing cancer-relevant comorbidities such as lung artery embolism and deep vein thrombosis [12, 13, 16, 19].

In terms of postoperative outcomes, this study found that hemorrhagic BM did not significantly impact intraoperative blood loss, surgical duration, or the risk of postoperative rebleeding. These findings suggest that the presence of hemorrhagic BM does not inherently signify a predisposition to general bleeding tendencies and that neurosurgical resection of hemorrhagic BM can be performed safely with appropriate perioperative management, including meticulous hemostasis and vigilant postoperative monitoring. Nonetheless, the occurrence of preoperative hemorrhage correlated with notably reduced overall survival, consistent with findings from prior studies [17]. Our data suggest that this outcome is not attributable to heightened complications during the removal of BM. Furthermore, additional research suggests comparable recurrence rates following the resection of both hemorrhagic and non-hemorrhagic BM cases [20]. Given the known association of ICH with heightened peritumoral edema, inflammation, and iron-induced oxidative injury, it is reasonable to speculate that these mechanisms may similarly pertain to hemorrhagic BM [21]. Taken together, currently available data alongside those of other studies, emphasize the prognostic significance of hemorrhagic patterns in BM patients.

The strengths of this study include its comprehensive analysis of preoperative predictors, perioperative management strategies, and postoperative outcomes in patients with hemorrhagic BM. Additionally, the inclusion of patients with diverse primary tumor origins enhances the generalizability of the findings. However, several limitations should be acknowledged, including the retrospective and single-center design, potential selection bias, and reliance on medical record data for outcome assessment. Future studies with larger cohorts and extended follow-up periods are necessary to validate our findings and assess the underlying reasons for the association between Intratumoral hemorrhage and diminished outcomes.

In conclusion, this study provides valuable insights into the risk factors and clinical implications of spontaneous hemorrhagic events in patients with BM. Our findings underscore the safety of anticoagulation in patients with BM and highlight the safety of neurosurgical treatment in patients with hemorrhagic BM and normal coagulation after pausing blood-thinning medication. We provide evidence suggesting decreased survival in cases of BM with hemorrhagic growth patterns.

## Conclusion

This study confirms the safety of anticoagulation in BM patients and the safety of neurosurgery for hemorrhagic BM. Melanoma metastases were notably linked to preoperative hemorrhage, while poor clinical status, impaired overall prognosis, and larger tumor volume predicted spontaneous bleeding. Hemorrhagic growth patterns were correlated with reduced survival and further research is necessary to validate these results and explore the underlying mechanisms driving this association.

### Electronic supplementary material

Below is the link to the electronic supplementary material.

Supplementary Material 1

## Data Availability

No datasets were generated or analysed during the current study.
