# Supplementary material for: Predictors and surgical outcome of hemorrhagic metastatic brain malignancies
Source: J Neurooncol. 2024 May 27;169(1):165–73. doi: 10.1007/s11060-024-04714-2 (PMC11269501; doi:10.1007/s11060-024-04714-2)
Supplement: Supplementary file 1 — Supplementary Material 1 [file 11060_2024_4714_MOESM1_ESM.docx]

| **Supplemental Table 1: Univariate analysis of laboratory parameters.** | | | | | | |
| --- | --- | --- | --- | --- | --- | --- |
| **Parameter** | **Reference** | **BM with hemorrhage**  **(N = 54)** | **BM w/o  hemorrhage**  **(N = 175)** | **p-value** | **OR** | **95%CI** |
| aPTT (s) | 24.4 – 32.4 | 24.39 ± 2.17 | 24.65 ± 2.60 | 0.499 ^a^ | N/A | N/A |
| PT (%) | 70 – 130 | 99.59 ± 13.01 | 101.96 ± 13.64 | 0.261 ^a^ | N/A | N/A |
| INR | < 1.1 | 1.00 ± 0.06 | 1.00 ± 0.08 | 0.544 ^a^ | N/A | N/A |
| Platelet count (/nL) | 140 – 320 | 310.26 ± 136.61 | 271.60 ± 97.12 | **0.022 ^a^** | N/A | N/A |
| Hb (g/dL) | 13.7 – 17.2 | 12.61 ± 2.61 | 13.11 ± 1.88 | 0.128 ^a^ | N/A | N/A |
| Hct (%) | 0.4 – 0.5 | 0.39 ± 0.10 | 0.37 ± 0.06 | 0.056 ^a^ | N/A | N/A |
| Univariate analysis of laboratory parameters associated with blood coagulation to detect preoperative differences between patients with or without BM-associated hemorrhage. All significant factors are highlighted in bold style. Annotations: ^a^ Student’s t-test or Mann-Whitney-U test. Abbreviations: aPTT, activated partial thromboplastin time; Hb, hemoglobin; Hct, hematocrit; INT, international normalized ratio; PT, prothrombin time. | | | | | | |
